# The inhibitory role of Mir-29 in growth of breast cancer cells

**DOI:** 10.1186/1756-9966-32-98

**Published:** 2013-12-01

**Authors:** Zhenglong Wu, Xiaona Huang, Xing Huang, Qiang Zou, Yujiang Guo

**Affiliations:** 1 Department of Surgery, Division of Thyroid and Mammary Surgery, Nanjing Medical University Affiliated Wuxi People’s Hospital, 299 Qingyang Road, Wuxi 214000, China; 2 Department of Medicine, Division of Oncology, Wuxi Traditional Medicine Hospital, 35 Houxixi Road, Wuxi 214000, China; 3 Department of Surgery, Division of Urology, Nanjing Medical University Affiliated Wuxi People’s Hospital, 299 Qingyang Road, Wuxi 214000, China; 4 Department of General Surgery, Fudan University Affiliated Shanghai Huashan Hospital, 12 Wulumuqi Zhong Road, Shanghai 200000, China; 5 Department of Surgery, Nanjing Medical University Affiliated Wuxi People’s Hospital, 299 Qingyang Road, Wuxi 214000, China

**Keywords:** Mir-29, Breast cancer growth, Apoptosis, Cancer progression

## Abstract

Mir-29 microRNA families are involved in regulation of various types of cancers. Although Mir-29 was shown to play an inhibitory role in tumorigenesis, the role of Mir-29 in breast cancer still remains obscure. In this study, we showed that Mir-29a is the dominant isoform in its family in mammary cells and expression of Mir-29a was down-regulated in different types of breast cancers. Furthermore, over-expression of Mir-29a resulted in significant slower growth of breast cancer cells and caused higher percentage of cells at G0/G1 phase. Consistent with this over-expression data, knockdown of Mir-29a in normal mammary cells lead to higher cell growth rate, and higher percentage of cells entering S phase. We further found that Mir-29a negatively regulated expression of B-Myb, which is a transcription factor associated with tumorigenesis. The protein levels of Cyclin A2 and D1 are consistent with the protein level of B-Myb. Taken together, our data suggests Mir-29a plays an important role in inhibiting growth of breast cancer cells and arresting cells at G0/G1 phase. Our data also suggests that Mir-29a may suppress tumor growth through down-regulating B-Myb.

## Introduction

Breast cancer is the most common cancer diagnosed in women. Although there were noteworthy advances in the early diagnosis and treatment during the past several decades, breast cancer still stands as the leading cause of cancer death in women worldwide [1,2]. The underlying mechanism for breast cancer development and metastasis is far from being completely understood. The high prevalence of this disease calls for more mechanistic insights for the development of new generation diagnostic and therapeutic strategies. Recently (after 2005), there is a growing interest in the roles of a new class of small non-coding RNAs, microRNAs (miRNAs) in breast cancer development [3,4].

MicroRNAs are ubiquitously expressed small RNAs which exert negative regulatory effects on gene expression at a post-transcriptional level [5]. Given the fact that microRNAs theoretically target any mRNA, it is likely that microRNAs possess a very broad functional spectrum which includes cell cycle regulation, cell growth, apoptosis, cell differentiation and stress response [5-9]. Consistent with this notion, it is no surprise that microRNAs are extensively involved in human cancer development [10]. To date, there are over 1000 miRNAs that have been discovered in human, among which MiR-29 stands as one of the most intriguing miRNA families which may play pivotal roles in cancer biology [8,11].

Composed of three mature members (MiR-29a, b and c), this family has been shown to be down-regulated in many different types of cancers and have been attributed predominantly tumor-suppressing properties. In lung cancers, MiR-29 family was reported to regulate specific genes associated with tissue invasion and metastasis in lung adenocarcinoma [8]. In hematologic neoplasms, MiRNA-29 expression levels are inversely correlated with prognosis of Mantle cell lymphoma (MCL) [12]. In addition, MiR-29 reduces cell growth and induces apoptosis in primary acute myeloid leukemia (AML) cells and related cell lines [13]. Moreover, it has been reported that by inhibiting MMP2 activity, MiR-29 plays an important inhibitory role in APOBEC3G induced colon cancer migration and invasion [14]. Finally, consistent with the data from studies on other types of cancer, MiR-29 family inhibits ovarian cancer development by targeting DNA methyltransferases 3A and 3B [15].

Unfortunately, there is relatively lack of information on the role of MiR-29 in breast cancer. Study from JK Richer’s group demonstrated that Mir-29a has an inhibitory role in tumor growth in vivo [16]. However, in another paper, the authors showed that MiR-29a may promote metastasis through facilitating epithelial-to-mesenchymal transition [17]. Thus, the function of Mir-29 in tumorigenesis and metastasis of breast cancer still remains unclear. In the current study, we are endeavored to further elucidate the roles of MiR-29 in breast cancers, which highlights MiR-29 as a potential new biomarker and therapeutic target for breast cancer.

## Materials and methods

### Reagents

Micro-RNA assays for mir-29a (002112), mir-29b (000413), mir-29c (000587) and RUN48 (001006) were purchased from Applied Biosystems. Fetal bovine serum (FBS) was from GIBCO. SuperSignal Substrate Western blotting detection system was from Pierce (USA). PVDF membrane was purchased from Bio-Rad Inc. B-Myb antibody (05–175) and cyclin D1 antibody were purchased from Millipore. Cyclin A2 (ab32498) antibody and GAPDH antibody (ab9485) were purchased from Abcam. Luciferase Assay Kit and pMIR-REPORT System were purchased from Applied Biosystems. β-Gal Assay Kit was purchased from Invitrogen (K1455-01). Lipofectamine 2000 reagent was purchased from Invitrogen.

### Cell culture

T-47D, MDA-MB-453, MCF-7 and MCF-10A cells were obtained from American Type Culture Collection. Human Mammary Epithelial Cells (HMEC) were purchased from Invitrogen (A10565). Cells were maintained in their proper media recommended by the companies and placed in a humidified incubator with 5% CO2 and 95% air at 37°C.

### Plasmids and transduction

A DNA fragment containing the hsa-miR-29a precursor (plus 100 bp upstream and 100 bp downstream) was amplified from genomic DNA of HMEC cells and cloned into pcDNA(+)3.1 vector (Invitrogen). The primers used here are: 5′-gaattcactcattccattgtgcctgg-3′ and 5′-ctcgagttgctttgcatttgttttct-3′.

MiRZip-29a construct (MZIP29a-PA-1) and its vector control (SI505A-1) were obtained from System Biosciences.

For the luciferase assay, pMIR-REPORT System (Applied Biosystems) was used. The plasmids (pMIR-REPORT-Luciferase-B-Myb-3′-UTR and its mutant) were constructed by following methodology. A 363-bp fragment (nt 2319–2681) of the 3′UTR of B-Myb (NM_002466.2) containing the miR-29a binding site was cloned into the pMIR-REPORT-Luciferase vector between HindIII and SacI sites. This fragment was amplified by PCR using the primers: gcgcaagcttggtgttgagggtgtcacgag and gcgcgagctctgcaccaagagagggtgagc. QuikChange Site-Directed Mutagenesis Kit (Stratagene) was used to generate pMIR-REPORT-Luciferase-B-Myb-3′-UTR-mutant plasmid by using following primers: 5′-ggctcctgagattaacaacaaa-3′ and 5′-tttgttgttaatctcaggagcc-3′. A plasmid coding β-galactosidase (pMIR-REPORT β-gal control) was used to normalize variability due to differences in cell viability and transfection efficiency.

### Cell transfection

MDA-MB-453 cells were transfected with vector or plasmid encoding hsa-miR-29a precursor by using lipofectamine 2000. After drug-selection (0.5 mg/ml G418 for 7 days), cells were used in different experiments. Transfection of MDA-MB-453 cells for luciferase assay is described in detail below.

### Packaging of pseudoviral particles and transduction of the target cells

MiRZip-29a plasmid or its vector control was transfected into 293TN cells and pseudoviral particles were collected following the provider’s protocol. Pseudoviral particles were applied on MCF-10A cells. 24 hours later, cells were subjected to drug selection (1 μg/ml puromycin) for 3 days. After drug-selection, cells were used in different experiments.

### Luciferase assay

To directly evaluate the effect of mir-29a on B-Myb, we used the luciferase assay. MDA-MB-453 cells were first transfected with vector or plasmid encoding hsa-miR-29a precursor. After drug-selection, cells were transfected with pMIR-REPORT-Luciferase-B-Myb-3′-UTR or its mutant using lipofectamine 2000. A plasmid encoding beta-galactosidase (pMIR-REPORT β-gal) was co-transfected with these plasmids. 48 hours later, luciferase activity was measured by using Luciferase Assay Kit following the manufactory protocol. Beta-galactosidase activity was measured by using β-Gal Assay Kit. The luciferase activity was normalized against the β-Gal activity from the same cells.

### Western blot

Proteins extracted from different cells were subjected to electrophoresis on a polyacrylamide gel and then transferred onto PVDF membranes. After that, membranes were blocked with 5% fat free dry milk in TBS-T for 1 h. The primary antibodies were applied on the membranes at 4°C overnight before they were washed out by TBS-T. The membranes were then incubated with secondary antibodies for 1 h at room temperature in TBS-T. After four washes in TBS-T, chemiluminescent substrate (Pierce, USA) was applied onto the membranes and the films were processed in a dark room.

### TaqMan miRNA analysis

The experiments were carried out following the manufactory protocol. Briefly, for RT reactions, 10 ng of total RNA was used in each reaction and mixed with the miRNA-specific RT primer. The thermal cyclers are as following: 16°C for 30 min, 42°C for 30 min, 85°C for 5 min. After the RT reaction, the products were diluted at 1:15, and 1.33 μl of the diluted RT-product was used for PCR reaction with specific primers. The thermal cyclers are as following: 95°C for 10 min, 95°C for 15 sec, 60°C for 60 sec, 40 cycles. The real-time PCR results were analyzed by using CT values. RUN48 was used for normalization.

### Guava assay

The experiments were carried out following the manufacture’s protocol. Briefly, cells were cultured in 6-well plates and harvested using standard protocols. Then cells were washed once with ice-cold PBS, fixed with 70% ethanol (−20°C) and stored at 4°C. Then the ethanol was removed and the cells were washed once with ice-cold PBS before staining. Finally, 200 μl Guava Cell Cycle reagent was used to resuspend about 2 × 10^5^ cells and cells were transferred to 96-well plates for data acquirement.

## Results

### Mir-29a is the dominant member of mir-29 family

**Figure 1:**
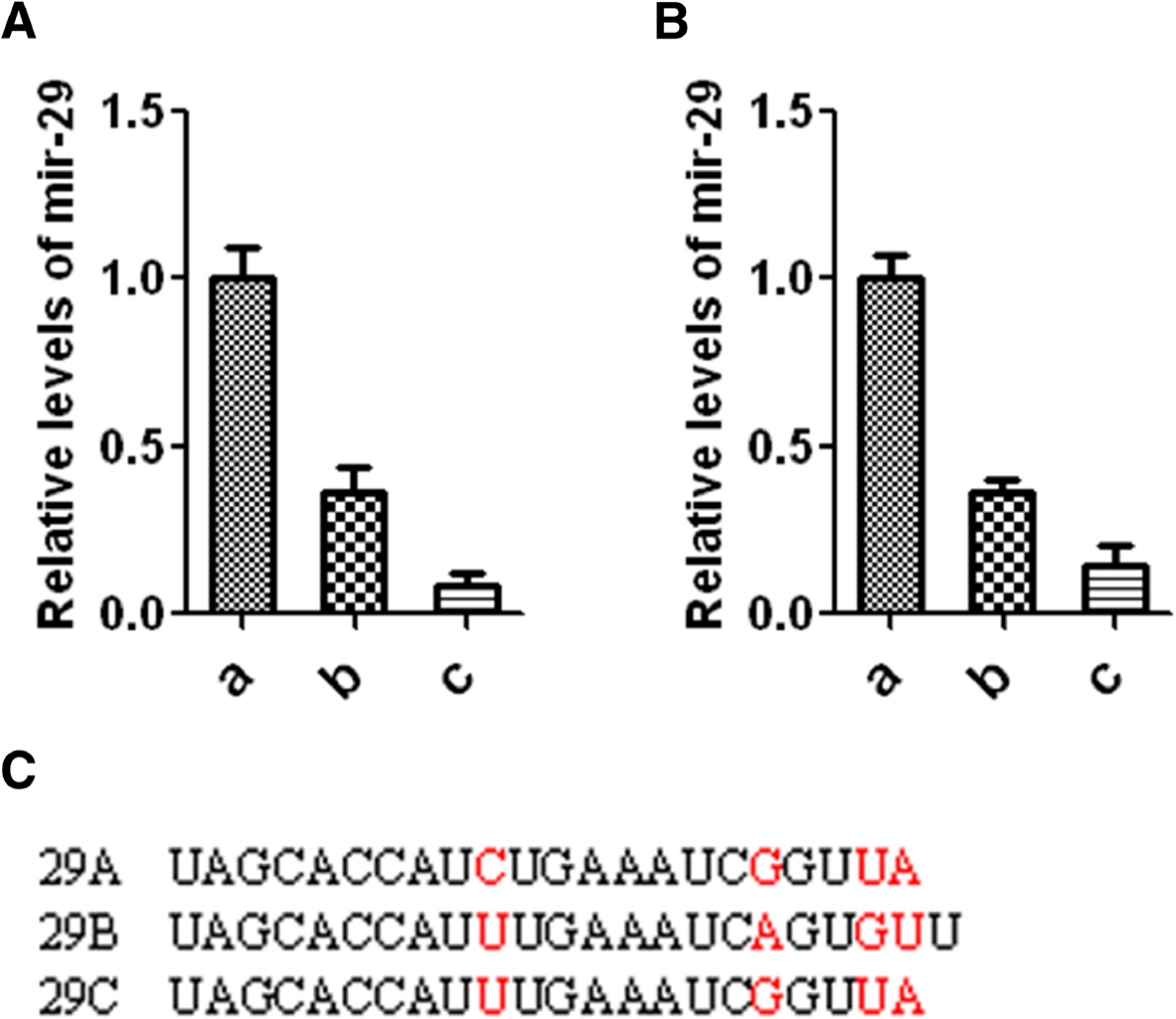
**The relative levels of mir29 isoforms in mammary epithelial cells. A**, the relative levels of mir29 isoforms in MCF-10A, n = 5, Mean ± SD. **B**, the relative levels of mir29 isoforms in HMEC, n = 5, Mean ± SD. **C**, the comparison of mir29 isoforms.

Mir-29 family is composed of three members Mir-29a, b and c, which are involved in tumorigenesis, chronic lymphocyte leukemia, acute myeloid leukemia and apoptosis [13,18]. In order to detect relative levels of three isoforms of Mir-29 family, Taqman MicroRNA assays were performed in MCF-10A and HMEC cells (Figure 1A and 1B). In both MCF-10A and HMEC cells, the expression levels of Mir-29a are significantly higher than the other two isoforms, indicating Mir-29a may play a more important role than the others. Because Mir-29a is the dominant isoform of Mir-29 family in mammary cells (>65% of total Mir-29 expression), and also due to the high similarity among three isoforms (Figure 1C), thus the following study mainly focuses on Mir-29a.

### Expression levels of Mir-29a are significantly lower in breast cancer cells when compared to those in normal mammary cells

**Figure 2:**
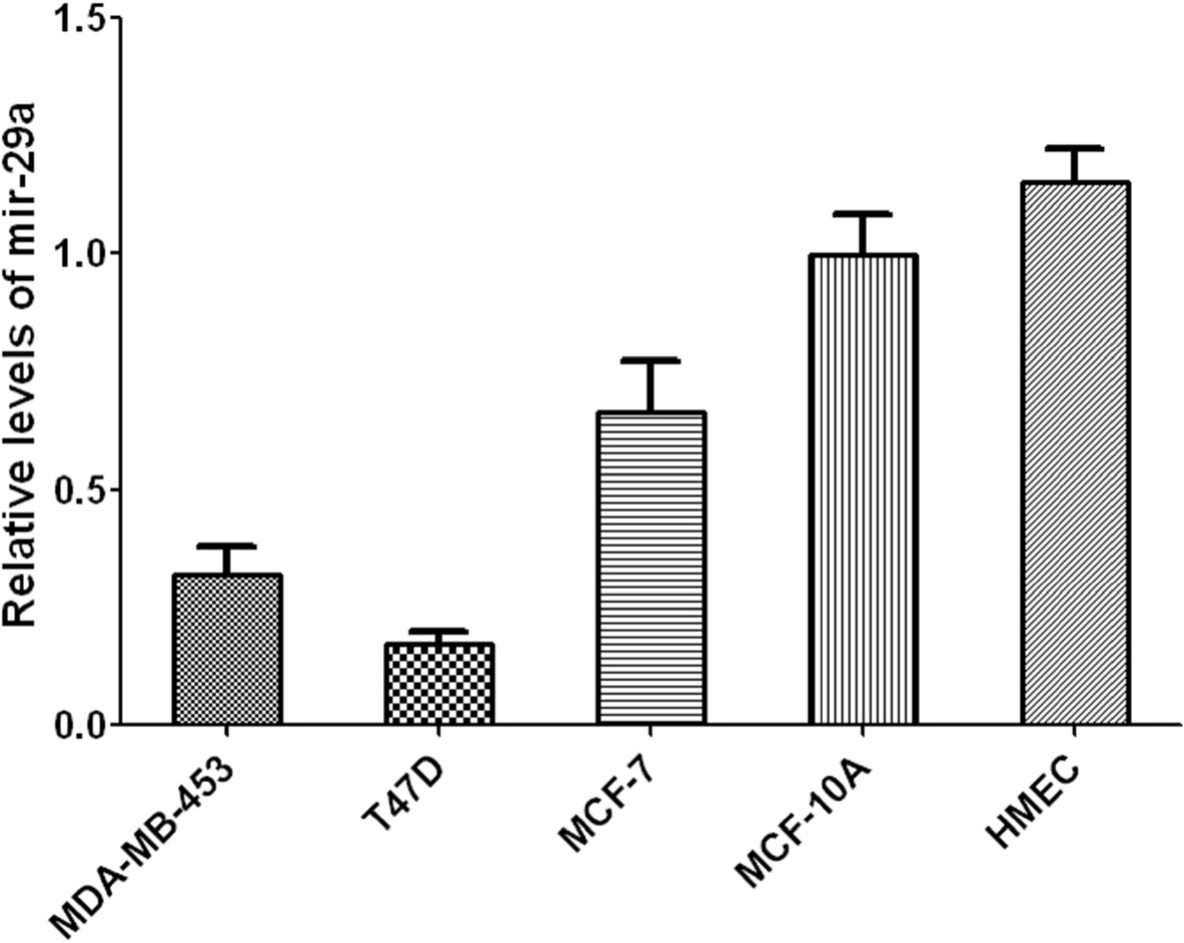
**Relative levels of mir-29a in normal mammary epithelia and breast cancer cells.** Here relative levels of mir-29a were normalized against that level in MCF-10A cells, n = 5, Mean ± SD.

Previous studies have showed that Mir-29 isoforms are involved in suppression of tumorigenesis [3,15,19-21]. Thus it is reasonable to hypothesize that expression of Mir-29a is altered in breast cancer cells, and over-expression of Mir-29a may suppress breast cancer cell growth. To test the hypothesis, expression levels of Mir-29a were assessed in normal human mammary epithelial cells (HMEC), immortalized normal breast epithelia (MCF-10A) and breast cancer cells (MDA-MB453, T47D and MCF-7) (Figure 2). As shown in Figure 2, expression levels of Mir-29a were significantly lower in breast cancer cells. Expression levels of Mir-29a decreased approximately by 83% in T47D cells, 68% in MDA-MB-453 and 33% in MCF-7 cells compared to expression level of Mir-29a in MCF-10A cells. The down-regulated expression level of Mir-29a in various breast cancer cell lines strongly suggests that Mir-29a is inhibitory to cancer cells.

### Over-expression of Mir-29a inhibits growth of MDA-MB-453 cells

**Figure 3:**
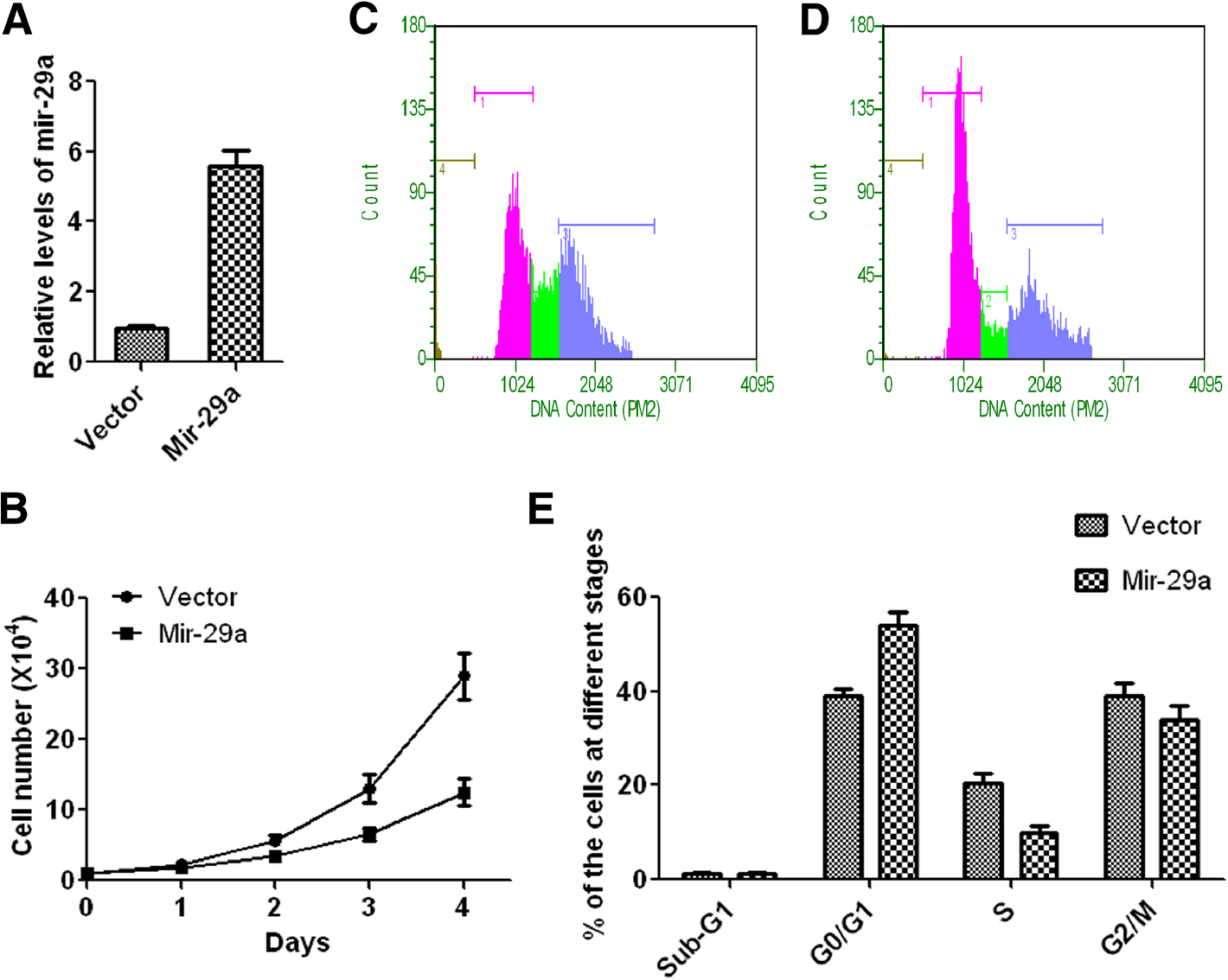
**Over-expression of miR-29a in MDA-MB-453 cells inhibits growth of cells. A**, relative levels of mir-29a in cells with or without mir-29a over-expression, n = 5, Mean ± SD. **B**, the growth curve of above cells, n = 5, Mean ± SD. **C** and **D**, representative figures of cell cycle analysis using Guava assay. **E**, quantitative analysis of the results of cell cycle examination, n = 5, Mean ± SD.

To further study whether Mir-29a negatively regulates cancer cell growth, Mir-29a was over-expressed in MDA-MB-453 cells. As shown in Figure 3A, Mir-29a expression level was 5.6-fold higher in cells transduced with Mir-29a over-expression construct than vector control. MDA-MB-453 cells over-expressed with Mir-29a displayed significantly slower growth rate than control cells (Figure 3B). To further determine if slower cell growth rate was due to perturbation of cell cycles progression, cell cycle profile was investigated by monitoring cell numbers at different stages (Figure 3C-E). Interestingly, compared to vector control, over-expression of Mir-29a caused 15% (P < 0.01) more cells to stay at G0/G1 phase (Figure 3E). This data suggested that over-expression of Mir-29 resulted in the arrest of cell cycle in G0/G1 phase and prevention of cells from entering into the S phase.

### Mir-29a knockdown facilitates growth of MCF-10A cells

**Figure 4:**
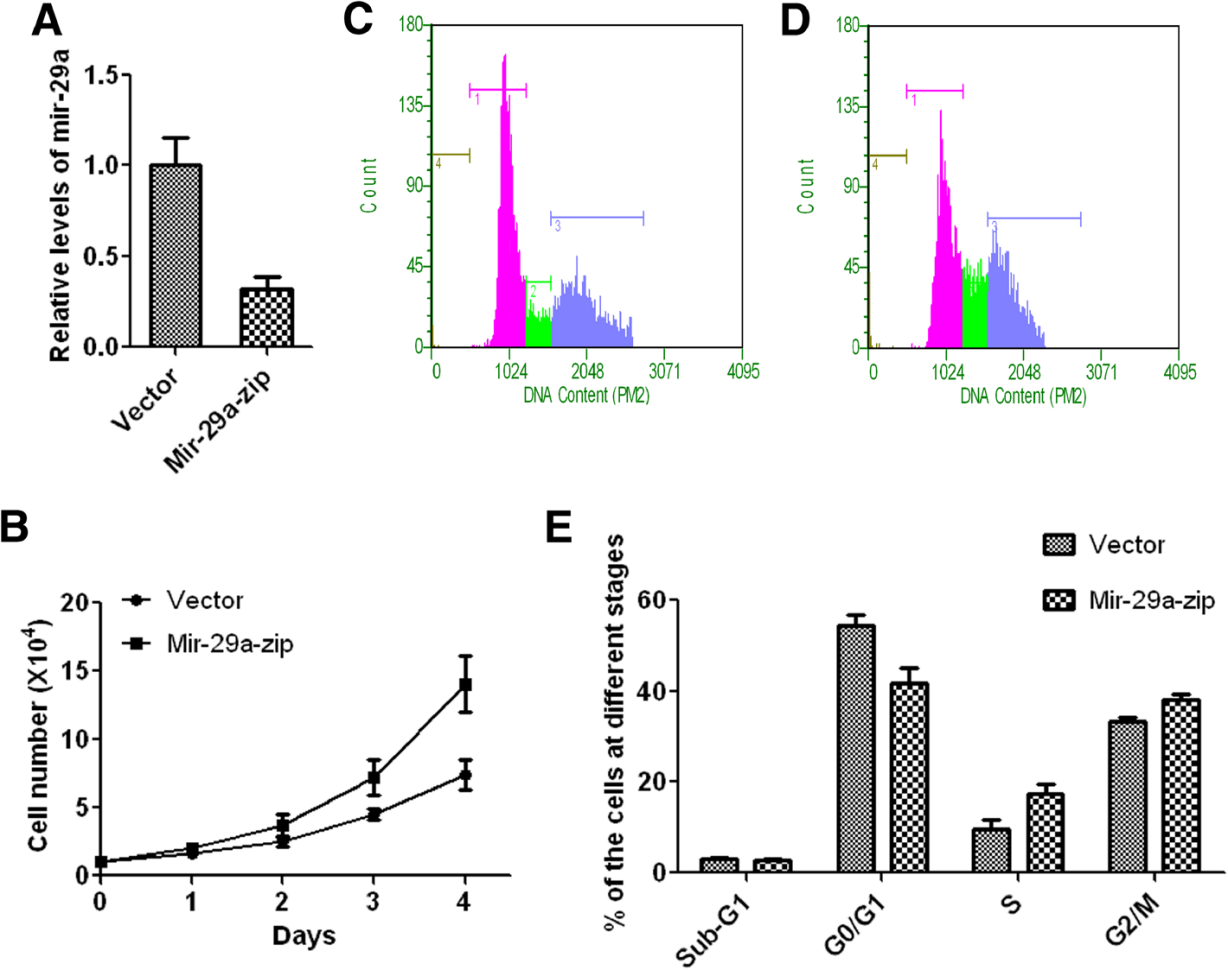
**Knockdown of miR-29a in MCF-10A cells increases growth of cells. A**, relative levels of mir-29a in cells with or without mir-29a knockdown, n = 5, Mean ± SD. **B**, the growth curve of above cells, n = 5, Mean ± SD. **C** and **D**, representative figures of cell cycle analysis using Guava assay. **E**, quantitative analysis of the results of cell cycle examination, n = 5, Mean ± SD.

To confirm the inhibitory role of Mir-29a, cell growth and cell cycle profile were investigated in MCF-10A cells with Mir-29a knockdown. Suppression of Mir-29a resulted in a higher cell growth rate than empty vector control (Figure 4A and 4B). In MCF-10A cells with knockdown of Mir-29a, the percentage of cells at G0/G1 phase was 12% (P < 0.01) lower than that in control cells (Figure 4C-E). This data suggested that knockdown of Mir-29a in normal cells caused more cells entering to S phase and thus promote cell growth. These results, together with data of over-expression of Mir29a in breast cancer cells, strongly suggested Mir-29a participates in arresting cells at G0/G1 phase and thus inhibiting tumor cell growth.

### Mir-29a negatively regulates cell growth through its depression on B-Myb expression

**Figure 5:**
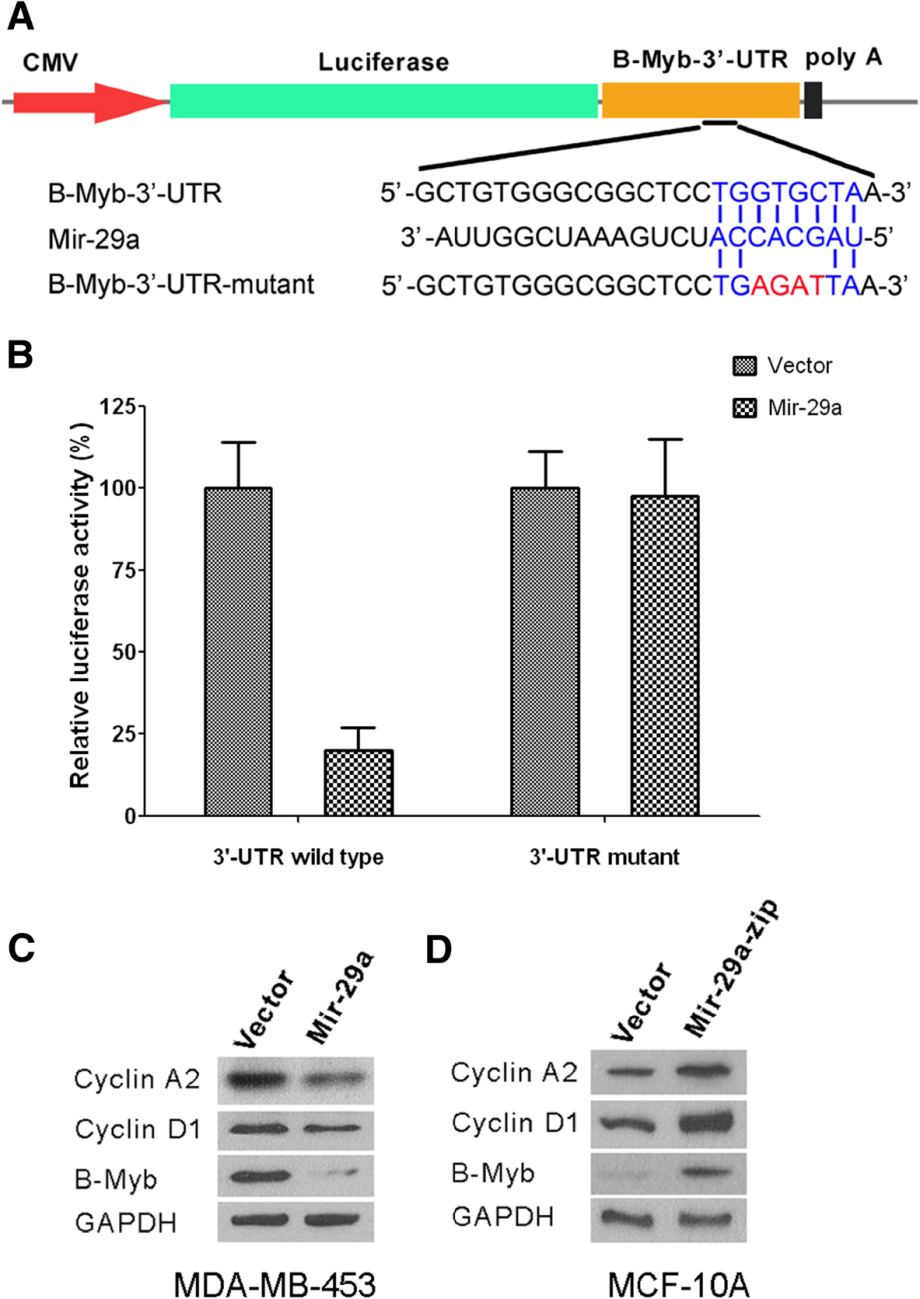
**B-Myb acts as the downstream effector of mir-29a to regulate cell cycle. A**, the scheme of the plasmid construction for the luciferase assay. **B**, relative luciferase activities of the cells (with or without mir-29a over-expression) transfected with either wild or mutant 3′-UTR of B-Myb; n = 5, Mean ± SD. **C**, protein levels of cyclin A2, cyclin D1 and B-Myb in MDA-MB-453 cells with or without mir-29a over-expression. **D**, protein levels of cyclin A2, cyclin D1 and B-Myb in MCF-10A cells with or without mir-29a knockdown.

The next question is how Mir-29a inhibits growth of cells. To further investigate this question, we searched the literature and found Mir-29a might inhibit growth of cells by down-regulating the transcription factor, B-Myb [22]. To evaluate the direct effect of mir-29a on B-Myb expression, we used pMIR-REPORT System. A fragment representing 3′-UTR of B-Myb was cloned into the 3′ end of luciferase gene (Figure 5A). As shown in Figure 5B, in mir-29a over-expressed cells, the expression of luciferase was dramatically inhibited (P < 0.01). In contrast with inhibition of mir-29a on wild type 3′-UTR of B-Myb, mir-29a cannot inhibit the luciferase expression (P > 0.05), when the binding site of mir-29a in 3′-UTR of B-Myb was mutated. Consistent with this, in MDA-MB-453 cells that over-expressed Mir-29a, protein level of B-Myb decreased (Figure 5C). Consistently in these cells, the downstream effectors of B-Myb such as Cyclin A2 and D1 were also down-regulated by Mir-29a over-expression (Figure 5C). On the contrary, in MCF-10A cells with Mir-29a knockdown, the protein level of B-Myb is dramatically up-regulated (Figure 5D). Consistent with an increased level of B-Myb, in MCF-10A cells, levels of Cyclin A2 and D1 were also up-regulated. All these findings suggested that Mir-29a probably regulates cell growth through B-Myb.

## Discussion

As described earlier, the function of Mir-29a in tumorigenesis and metastasis remains controversial. Muniyappa *et al.* showed that Mir-29a was down-regulated in invasive lung cancer cells and invasive phenotype of cancer cells could be suppressed by ectopic expression of Mir-29a [23]. Study from Xu et al. also showed that expression level of Mir-29a is significantly lower in various solid tumors [24]. In contrast, Mir-29a is also shown to be up-regulated in certain leukemia cells [25]. In this study, we focused on the role of Mir-29a in breast cancers cells. We showed that expression level of Mir-29a is down-regulated in various breast cancer cells (Figure 2). This data indicates that Mir-29a expression is probably associated with breast cancer. One piece of evidence to support this hypothesis is that over-expression of Mir-29a in breast cancer cells significantly reduce cancer cell growth rate (Figure 3B). Consistent with this result, knockdown of Mir-29a in normal mammary epithelial cells cause higher cell growth rate (Figure 4B). These data strongly suggested Mir-29a inhibited tumorigeneses through suppression of cell growth. We also showed that the inhibitory effect of Mir-29a to breast cancer cells is probably due to its role in arresting cells in G0/G1 cells (Figure 3C-E and 4C-E). Previous studies showed that Mir-29a is able to suppress the expression of tristetraprolin, which is involved in epithelial-to-mesenchymal transition [17]. A study also showed that Mir-29a targets protein P42.3, which was also found, associated with tumorigenicity [26]. In this study, we showed that Mir-29a negatively regulated expression of B-Myb (Figure 5), which is a transcription factor broadly involved in regulating cell cycle and apoptosis and probably is a promoting factor for cancer [27]. Downstream effectors of B-Myb, such as Cyclin A2 and D1, were also correspondingly regulated by Mir-29a. Cyclin D1 is one of highly over-expressed proteins in breast cancer cells and over-expression of Cyclin D1 protein was found in 40-90% of cases of invasive breast cancer [28]. Cyclin A2 is involved in S phase and G2-M phase transition and is also over-expressed in various cancers [29-31]. Taken together, in current paper, we showed that Mir-29a may act as a tumor suppressor through its inhibitory function on growth of breast cancer cells, and down-regulating expression of B-Myb by Mir-29a may contribute to this process.

## Competing interests

The authors declare that they have no competing interests.

## Authors’ contributions

ZW and XH designed the study, performed the experiments except the Guava assay and drafted the manuscript. XH performed the Guava assay. QZ provide technical support on experimental design, help to conduct the Guava assay and important comments in improving the manuscript. YG designed the study, drafted the manuscript and interpret the data. All authors read and approved the final manuscript.
